# Preparation of Renewable Bio-Polyols from Two Species of *Colliguaja* for Rigid Polyurethane Foams

**DOI:** 10.3390/ma11112244

**Published:** 2018-11-11

**Authors:** Diana Abril-Milán, Oscar Valdés, Yaneris Mirabal-Gallardo, Alexander F. de la Torre, Carlos Bustamante, Jorge Contreras

**Affiliations:** 1 Departamento de Biología y Química, Facultad de Ciencias Básicas, Universidad Católica del Maule, 3460000 Talca, Chile; dabril@ucm.cl; 2 Vicerrectoría de Investigación y Postgrado, Universidad Católica del Maule, 3460000 Talca, Chile; 3 Instituto de Ciencias Químicas Aplicadas, Facultad de Ingeniería Civil, Universidad Autónoma de Chile, 3460000 Sede Talca, Chile; yaneris.mirabal@uautonoma.cl; 4 Departamento de Química Orgánica, Facultad de Ciencias Químicas, Universidad de Concepción, Casilla 160-C, 4030000 Concepción, Chile; afernandezd@udec.cl; 5 Facultad de Ciencias Agrarias y Forestales, Universidad Católica del Maule, 3460000 Talca, Chile; cbustamantevaldes@gmail.com (C.B.); jcontrer@ucm.cl (J.C.)

**Keywords:** *Colliguaja integerrima*, *Colliguaja salicifolia*, vegetable oil, bio-polyol, renewable resources

## Abstract

In this study, we investigated the potential of two non-edible oil extracts from seeds of *Colliguaja integerrima* (CIO) and *Colliguaja salicifolia* (CSO) to use as a renewable source for polyols and, eventually, polyurethane foams or biodiesel. For this purpose, two novel polyols from the aforementioned oils were obtained in a one-single step reaction using a mixture of hydrogen peroxide and acetic acid. The polyol derivatives obtained from the two studied oils were characterized by spectral (FTIR, ^1^H NMR, and ^13^C NMR), physicochemical (e.g., chromatographic analysis, acid value, oxidizability values, iodine value, peroxide value, saponification number, kinematic viscosity, density, theorical molecular weight, hydroxyl number, and hydroxyl functionality) and thermal (TGA) analyses according to standard methods. Physicochemical results revealed that all parameters, with the exception of the iodine value, were higher for bio-polyols (CSP and CIP) compared to the starting oils. The NMR, TGA, and FTIR analyses demonstrated the formation of polyols. Finally, the OH functionality values for CIP and CSP were 4.50 and 5.00, respectively. This result indicated the possible used of CIP and CSP as a raw material for the preparation of polyurethane rigid foams.

## 1. Introduction

The high demand for products from petrochemical origin and their negative effects on the environment, parallel to the growing scarcity of these non-renewable resources, are factors that have encouraged the chemical industry to look for new sources of renewable resources as raw materials. These raw materials have contributed in a very positive way to the sustainable development of the plastics industry, due to the great synthetic potential of nature and different principles of green chemistry [1,2]. Vegetable oils are one of the most considered alternatives due to abundance, low toxicity, biodegradability, inherent fluidity, and low cost [3,4,5]. Several vegetable oil molecules must be chemically transformed to form polyols, and these bio-polyols are used for the obtention of polyurethanes [6], polyesters [7], and epoxy [8], among others.

The synthesis of bio-polyols from fatty acids and vegetable oils has been the focus of many studies. It is important to note that vegetable oils, with the exception of castor and lesquerella oils, do not contain hydroxyl groups. For that reason, it is necessary to chemically modify the vegetable oils to introduce hydroxyl groups into their structures to produce polyols. There are five different pathways for vegetable oil-based bio-polyol production: (1) epoxidation and oxirane ring-opening [9]; (2) hydroformylation and hydrogenation [10,11]; (3) ozonolysis [12]; (4) thiol–ene coupling [13]; and (5) transesterification/amidation [14]. The first method is the most widely used. Researchers regularly use this method in one or two steps. “The one-step reaction consists of the in situ epoxidation followed by hydroxylation using acetic and sulfuric acids and hydrogen peroxide. The two-step reaction deals with the triglyceride epoxidation followed by the ring-opening of oxirane, based on the use of difunctional molecules such as alcohols or amines” [15,16]. This multi-step synthesis would unquestionably increase the cost of bio-polyol production.

It is important to mention that various vegetable oils, including palm, soybean, sunflower, rapeseed, and canola oils, are used with slight modifications to produce polyols [17,18,19,20,21,22]. The great inconvenience with some of these oils are their use in food, which bring, as a consequence, a global imbalance to the food supply and demand in the industrial market [23].

Therefore, this study concentrates in the obtention of new bio-polyols from two non-edible oil extracts from seeds of *Colliguaja integerrima* and *Colliguaja salicifolia* by a single one-step reaction using a mixture of hydrogen peroxide and acetic acid. In addition, we characterized the prepared oils and polyols using various physicochemical, spectroscopic, and thermoanalytical methods.

*C. integerrima* and *C. salicifolia* are two varieties of the Euphorbiaceae family. The Euphorbiaceae family, with around 300 genera and over 7000 species, is one of the largest and most diverse families of flowering plants [24]. Both species grow wild in South America, specifically in the phytogeographical provinces of Patagonia and Monte in Argentina and Chile [25,26,27]. The oil extracted from these two species are known for the diuretic activity of their aqueous extract [28]. To our knowledge, no studies have been previously reported about the production of polyols from *C. integerrima* and *C. salicifolia* using a cheap and environmentally benign method.

## 2. Experimental Procedures

### 2.1. Chemicals and Reagents

Diethyl ether (C_4_H_10_O), *n*-hexane, glacial acetic acid (AcOH), hydrogen peroxide (H_2_O_2_), toluene (C_7_H_9_), sulfuric acid (H_2_SO_4_), sodium sulfate (Na_2_SO_4_), sodium carbonate (Na_2_CO_4_), sodium thiosulfate pentahydrate, and potassium hydroxide (KOH) were supplied by Arquimed (Santiago, Chile). Iodine monochloride solution (Wijs reagent), starch solution, tetrahydrofuran (THF), and phenolphthalein reagent were purchased from Sigma-Aldrich (St. Louis, MO, USA). All used reagents were of analytical grade, with the exception of *n*-hexane and tetrahydrofuran (HPLC purity grade). For GC-MS analysis, Supelco^®^ 37 component fatty acid methyl ester (FAME) mix in dichloromethane (varied concentrations) was purchased from Sigma-Aldrich.

### 2.2. C. integerrima and C. salicifolia Seeds

The seeds of *C. salicifolia* and *C. integerrima* were collected in Cayurranquil, a geographic area in the Cauquenes Province (725468, 6017270) and the Pehuenche International Pathway, Los Cóndores, at the foothills of Talca (348271, 6026739) Chile, respectively.

### 2.3. Vegetable Oil Extraction

Oil extraction was performed according to the AOAC method Am2-93 [29]. About 250 g of *C. integerrima* seeds were extracted using *n*-hexane (150 mL) as an extraction solvent in a Soxhlet apparatus. After 8 h, the *n*-hexane was removed by distillation under reduced pressure at 40 °C. The obtained *C. integerrima* orange oil (CIO) was stored at 4 °C under inert atmosphere until further investigation. The extraction was performed in triplicate for each harvested sample, obtaining a 26% yield. The same procedure was used for the *C. salicifolia* but, in this case, the oil (CSO) obtained had a yellow coloration with 23% of yield.

### 2.4. Synthesis of C. integerrima and C. salicifolia Polyol

The synthesis of the polyols was carried out following the methodology described by Monteavaro et al. [30] with slight modifications. A solution of 5 g (5.6 mmol) of *C. integerrima* oil and 9.30 mL (0.162 mol) of glacial acetic acid in 20 mL of toluene, with some drops of sulfuric acid, were placed into a 3-necked flask equipped with a mechanical stirrer, reflux condenser, and isobaric funnel. The mixture was mechanically stirred at room temperature until complete homogenization. After that, a solution of 30% H_2_O_2_ (5.30 mL) was slowly added controlling the temperature. When the H_2_O_2_ addition was completed, the mixture was heated to 60 °C for 12 h. Then, the reaction mixture was cooled to room temperature and a 10% (w/v) sodium bisulfide solution was added, and the mixture was stirred for 20 min to eliminate excess peroxide. After that, 50 mL of ethyl ether was added to the mixture, and the organic phase was washed several times with 10% (w/v) sodium carbonate solution to neutral pH. Finally, the organic phase was dried over sodium sulfate, and concentrated under vacuum to eliminate the ethyl ether to obtain the *C. integerrima* polyol (CIP). The same procedure was used for the synthesis of *C. salicifolia* polyol (CSP).

### 2.5. Characterization Methods Used for Oils and Polyols

The *C. integerrima* and *C. salicifolia* oils and polyols were characterized determining the fatty acid composition, the oxidizability value (Cox), acid value (AV), iodine value (IV), saponification number (SN), kinematic viscosity, peroxide value (PV), density, hydroxyl number, thermogravimetric analysis (TGA), Fourier transform infrared (FTIR), and ^1^H and ^13^C NMR spectroscopy. It is important to note that the fatty acid composition, peroxide value, and the oxidizability value were performed only for the studied oils, and the rest of the characterization techniques were done for the *C. integerrima* and *C. salicifolia* oils and polyols.

The fatty acid profile was determined only for the *C. integerrima* and *C. salicifolia* oils as fatty acid methyl esters by gas chromatography-mass spectrometry. The methyl esters were prepared using the method described by Morrison and Smith [31]. The separation of the fatty acid esters was performed using QP 5000 Shimadzu (Kyoto, Japan) gas chromatographer with mass spectrometer and autosampler was used, as well as the 1.2 Class-5000. A fused-silica column, coated with the DB-5 stationary phase, was utilized (30 m × 0.2 mm inner diameter, dry film thickness of 0.25 µm, J & W Scientific). The initial oven temperature was 60 °C, which was kept for 5 min; a 2 °C min^−1^ temperature increase was programed until it reached 220 °C; this temperature was kept for 30 min. The injector temperature was 220 °C. Helium was used as a carrier gas with a 1.0 mL min^−1^ flow. The injection volume was 1 µL (1% solution in CH_2_Cl_2_) with a 1:10 split ratio. Column pressure was 100 kPa. Mass detector conditions were the following: source temperature, 240 °C; electron impact mode (EI), 70 eV; scan rate of 1 scan s^−1^; and acquisition range, 29–450 u. Components were identified by comparing retention times related to a linear standard made with Supelco^®^ 37 Component FAME Mix in dichloromethane (varied concentrations) of an alkane series (C9–C24) and their mass spectra to those from the Wiley 330000 database and the ones reviewed from the literature. In addition, with the percentage of unsaturated fatty acids, we calculated the oxidizability values (Cox) of *C. integerrima* and *C. salicifolia* oils, applying the formula proposed by Fatemi et al. [32]:

(1) $$Cox=\frac{\left[1\left(16:1\%+18:1\%+20.1\%\right)+10.3\left(18:2\%+20:2\%\right)+21.6\left(18:3\%\right)\right]}{100}$$

The acid value, iodine value, peroxide value, and saponification number of the obtained samples (*C. integerrima* and *C. salicifolia* oils and polyols) were determined according to the AOCS Official Method Cd 3d-63, AOAC Official Method 920.158 (Hanus method), AOAC Official Method 965.33 and the AOAC Official Method 920.160, respectively [33,34]. The theorical molecular weights of both polyols were calculated using the formula proposed by Zlatanić et al. [35].

The structure of samples (*C. integerrima* and *C. salicifolia* oils and polyols) were analyzed by ^1^H and^13^C NMR, and FTIR spectroscopy, combined with hydroxyl number measurement. ^1^H and ^13^C NMR spectra were conducted with a Varian Inova 300 (Varian, Inc., Palo Alto, CA, USA), 300 MHz, using CDCl_3_ as a solvent and tetramethylsilane as an internal reference. FTIR spectra were recorded using an Agilent Cary 360 FTIR in the range of 4000 to 650 cm^−1^ at a resolution of 4 cm^−1^ with 32 scans on ATR (Agilent Technologies, Palo Alto, CA, USA). Two milliliters of the *C. integerrima* and *C. salicifolia* oil and polyol samples were put directly on the equipment plate without any previous treatment. The hydroxyl number of the *C. integerrima* and *C. salicifolia* polyols was determined according to ASTM 4274-05 [36]. The viscosity of the *C. integerrima* and *C. salicifolia* oil and polyol samples were measured by Brookfield viscometer (LVDV-II, Brookfield Engineering Laboratories, Inc., Stoughton, Massachusetts) at 25 ± 0.5 °C. The density of the *C. integerrima* and *C. salicifolia* oil and polyol samples were measured by means of a pycnometer at 25 ± 0.5 °C. Finally, thermal stability of the oils and polyols were performed in a thermogravimetric analyzer NETZSCH TG 209F1 Iris (NETZSCH Company, Germany) with 10 °C min^−1^ constant heating rate. The heating was from 30 °C to 500 °C in inert atmosphere with a flow rate of 60 mL min^−1^.

## 3. Results and Discussion

### 3.1. Physicochemical Characteristics of Vegetable Oils

The chemical composition and characteristics of the precursor of polyols (*C. integerrima* and *C. salicifolia* oils) used in this study are shown in Table 1. In addition, we determined the acid value, cox value, iodine value, peroxide value, saponification number, kinematic viscosity, and density of these oils.

**Figure 1:**
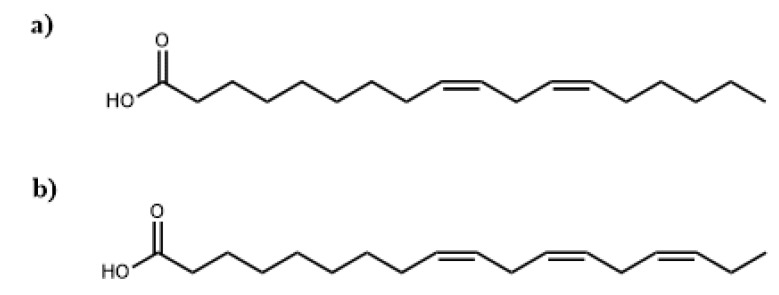
Chemical structures of linoleic (**a**) and linolenic (**b**) acids found in *C. integerrima* oil (CIO) and *C. salicifolia* oil (CSO).

As can we see, the saturated fatty acids (SFAs) of the oils differ very little, because the percentage of palmitic acid, which is the main SFA, is similar in the two studied oils. On the other hand, the percentages of unsaturated fatty acids (UFAs), were 87.36and 88.23% for CIO and CSO, respectively. Specifically, the dominant constituents (over 80%) of both studied oils are C18 fatty acids, in spite of there also being about 7% of C20 fatty acids. The percentage value of linoleic acid (C 18:2) was observed in 31.11% and 20.40% for CIO and CSO, respectively. The main differences between CIO and CSO are derived from the percentage of linolenic acid (C 18:3), observed in 26.39 and 46.60, respectively. Figure 1 shows the chemical structures of linoleic and linolenic acids found in CIO and CSO. It is important to note that this difference in the distribution of the double bonds in the fatty acid chains is fundamental for knowing the possibilities of increasing OH groups in the polyols synthetized using these oils, and directly proportional to their potential as a future polyurethane foam.

Taking into account the abovementioned and using the measured composition (lipid profile), we measured the degree of unsaturation by the iodine value (IV), which is directly related to the Cox value for CIO and CSO. The results for CIO and CSO indicated values of IV (143.8 and 179.3 g I_2_/100 g, respectively) and Cox (9.35 and 12.46, respectively), providing it a particular resistance to oxidation. The saponification value (SN), which is a parameter related with the molecular mass of the fatty acids found in the oil, was also studied. The SN values for CIO and CSO were 196 and 194 mg KOH/g oil, respectively, which are in the average SN range of 175–250 mg/g reported for common vegetable oils [37]. It should be noted that these values do not show significant differences, which means that the fatty acid composition of the studied oils are similar in molecular weight. It is known that acid value (AV) measures the number of carboxylic acid groups present in fat or oil; this value must not be too high, because it is a result of the hydrolysis of triglycerides. The highest acid value was found for CSO (0.25 mg KOH/g oil), however, the AV for both studied oils was less than 1.0 mg KOH/g oil (See Table 1), indicating that oils did not undergo hydrolytic processes. Additionally, the peroxide value (PV) of CIO and CSO were similar, and less than 20 meq/kg, respectively, indicating that these oils were unoxidized and of high initial quality.

Finally, we also measured the kinematic viscosity and density for CIO and CSO. Generally, the kinematic viscosity is related to a measure of the internal friction or resistance of an oil to flow. According to Krisnangkura et al. [38], viscosity may be considered the interaction forces of molecules. The viscosity values shown in Table 1 were 58.6 and 48.8 mm^2^/s for CIO and CSO, respectively. The difference in these values is due to the degree of unsaturation presented in both studied oils, and is in concordance with the results reported by Rodrigues Jr et al. [39]. In this research, Rodrigues Jr reported that one double bond increased the viscosity, whereas two or three double bonds caused a decrease in the viscosity of the systems. On the other hand, density is an important physical characteristic of any substance, and is the weight of a unit volume of fluid [40]. In this case, the values reported in Table 1 are 0.884 and 0.896 g/cm^3^ for CIO and CSO, respectively. It is important to know that all reported values shown in Table 1 (physical characteristics) of CIO and CSO had a drastic change in their numerical values when the oils became their respective polyols.

### 3.2. Synthesis and Characterization of C. integerrima and C. salicifolia Polyols Prepared by a One-Step Synthesis

The main goal of our research was to prepare polyols from CIO and CSO, respectively. *C. integerrima* and *C. salicifolia* polyols (CIP and CSP, respectively) were prepared by a one-step synthesis using the acetic acid/H_2_O_2_ system. The results reveal a wide difference of physicochemical characteristics among both obtained polyols and their starting oils. The measures of OH functionality was given an idea of the characteristics of the polyol obtained. For example, if the reaction was complete we obtained a polyol with high OH functionality. On the contrary, if the reaction was partial we obtained a polyol with remaining epoxy groups. These two results will be depending on the reaction conditions. As previously mentioned, we obtained CIP and CSP with OH numbers of 225.0 and 240.8 mg KOH/g (See Table 2), respectively. According to these values, it is evident that both hydroxylation reactions to obtain the CIP and CSP were complete, which are coherent with the NMR and FTIR results.

#### 3.2.1. ^1^H and ^13^C NMR Analysis

**Figure 2:**
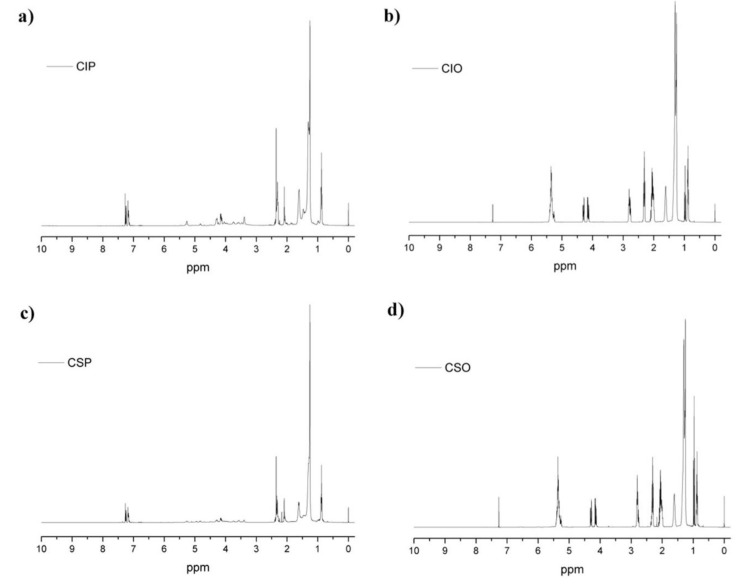
^1^H NMR spectra of CIP (**a**), CSP (**c**) whit their respective CIO (**b**) and CSO (**d**).

**Figure 3:**
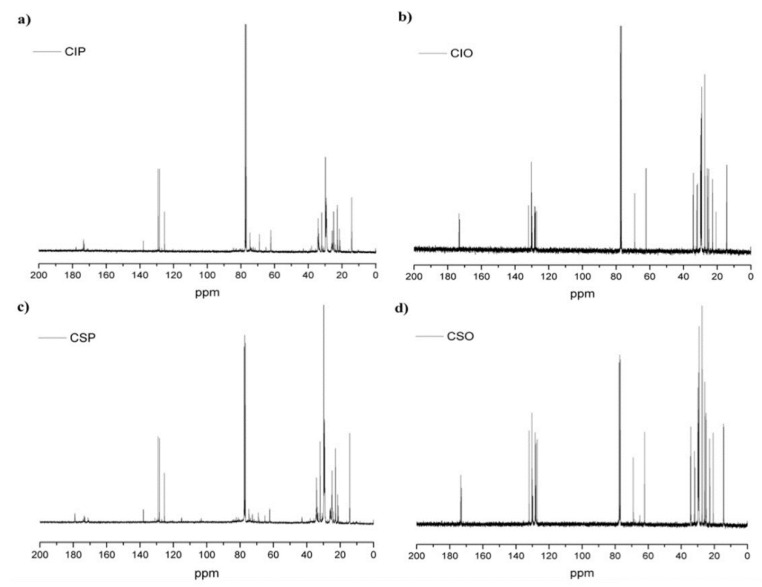
^13^C NMR spectra of CIP (**a**); CSP (**c**) with their respective CIO (**b**) and CSO (**d**).

**Figure 4:**
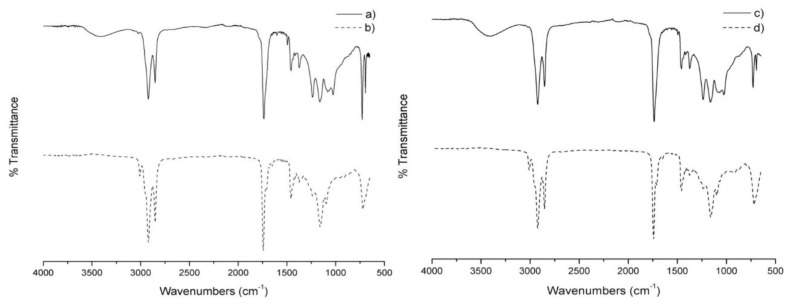
FTIR spectra of CIP (**a**); CSP (**c**) whit their respective CIO (**b**) and CSO (**d**).

The functional group present in the CIP and CSP, with their respective starting oils, was confirmed by ^1^H NMR, ^13^C NMR, and FTIR analyses, and the results are shown in Figure 2, Figure 3 and Figure 4, respectively. It is known that polyols are a complex blend of products. For that reason, the NMR spectra analysis was performed by zone, because each zone gives indications about the average structure of polyols.

Figure 2a,c shows the ^1^H NMR spectra of CIP and CSP, respectively. The main difference between polyols and their corresponding oils (Figure 2b,d) in the ^1^H NMR spectra were the appearance of new peaks in the zone between 3.27 and 3.83 ppm, which are assigned to the presence of methylene protons attached to the hydroxyl group (–CH–OH). In addition, the signal corresponding to the olefinic hydrogen (–CH=CH–) that appeared in the zone between 5.32 and 5.44 ppm disappeared, suggesting that polyol structures were practically without unsaturation. However, the signal corresponding to the methynic hydrogen of the glycerol moiety between 5.18 and 5.30 ppm (ROOCH_2_)_2_CH(OOR) remained. Finally, the absence of signals in the zone between 2.8 and 3.2 ppm, relative to the epoxide groups (–CH(O)CH–), confirmed the occurrence of the hydroxylation reaction to obtain the CIP and CSP.

On the other hand, the ^13^C NMR spectra of CIP and CSP with their respective starting oils offer similar information (See Figure 3). These spectra were characterized by the almost disappearance of the ethenic double bond signs of both starting oils, and the appearance of a new sign in 75.32 ppm, which corresponds to –CH–OH groups formed during the epoxy-opened ring. In agreement with the results obtained by the ^1^H NMR spectra, the signals centered in 54.38 ppm, relative to the epoxide groups nonappearance, proved the formation of polyols. Furthermore, the signal found at 173–175 ppm, relative to the carbonyl ester, showed a peak present in both oils and polyols.

Finally, it is important to mention that, in both polyol spectra, appear signals corresponding to the residual toluene used in the synthesis process. The signals corresponding to the toluene structure were found at δ 21.4, 125.2, 128.3, 129.2 ppm, and 2.35, 7.0–7.3 ppm for ^13^C NMR and ^1^H NMR, respectively. This evidence confirmed that the elimination of toluene was ineffective.

#### 3.2.2. FTIR Analysis

In order to complement the NMR analysis, the successful conversion of CIO and CSO into polyols was confirmed qualitatively by FTIR spectroscopy. These spectra are compared in Figure 3a–d, respectively. Figure 4a,c shows the FTIR spectra of CIP and CSP, respectively. Both polyols’ FTIR spectra exhibited a broad peak centered approximately at 3400 cm^−1^, which were assigned to the presence of a hydroxyl (–OH) stretching vibration. Another difference between CIP and CSP with their respective oils was the most intensive band (C=O, carbonyl stretching), attributed to the presence of the ester linkage. It slightly shifted from 1748.0 to 1738.8 cm^−1^ for CIO and CIP, respectively. The same behavior was found for CSO and the CSP, in this case. the band slightly shifted from 1743.7 to 1734.3 cm^−1^, respectively. Another important asymmetrical stretching band present in both FTIR spectra polyols corresponded to C–O–C groups at 1158 cm^−1^. It is important to mention that the absence of the bands at around 870 and 920 cm^−1^, assigned to the epoxy groups in both spectra polyols, corroborate that the hydroxylation reaction of the oils was complete. Finally, the bands at 2919.6 and 2846.1 cm^−1^, and 2924.3 and 2850 cm^−1^, assigned to asymmetrical and symmetrical stretching CH groups for CIP and CSP, respectively, were also detected. This evidence from FTIR analysis confirmed the formation of polyols, and the results obtained by ^1^H and ^13^C NMR.

#### 3.2.3. TGA Analysis

**Figure 5:**
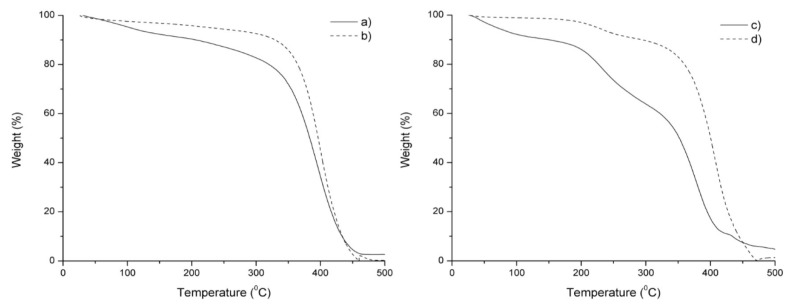
Thermogravimetric analysis (TGA) curve of CIP (**a**); CSP (**c**) with their respective CIO (**b**) and CSO (**d**).

In order to investigate the influence of the structure and composition on thermal stability of the oils and polyols, thermogravimetric analysis was performed in the interval from 30 to 600 °C. The TG curves are shown in Figure 5, respectively. Following the same analysis used for NMR and FTIR discussion, we compare the thermograms obtained for polyols and their respective oils. Analyzing the curves in Figure 5, the weight loss curves for the thermal degradation of both polyols closely resembles the TG curves of their starting oils. It is important to note that CIO and CSO are more thermally stable than their respective polyols. This behavior is probably related to the binding energy of the unsaturated chains functionalized during hydroxylation reactions. Specifically, the C=C and CH bonds present in the oils have binding energies of 614.2 and 413.4 kJ⋅mol^−1^, respectively, which enables them to be more thermally stable than the CO bond present in polyols, which has a lower binding energy (353.5 kJ⋅mol^−1^) [41].

According to the TG curves shown in Figure 4a and c, CIP and CSP exhibited slightly less thermal stability compared to their oils. In addition, the initial weight loss observed under 100 °C in the TG curve, in both polyols, could be attributed to the loss of the residual toluene molecules, which, as commented before, was used in the polyol synthesis. In the oils at 100 °C, this weight loss was not observed. The total weight loss for both polyols was about 95%, meanwhile, the oils degraded completely [42]. We suggest that these results could be due to the presence of hydroxyl groups in the polyol.

The TG curve of CIP indicates another degradation stage (Figure 5a) at 400.24 °C. The degradation mechanism probably corresponded to saturated and unsaturated fatty acid decomposition. On the other hand, a quite different decomposition was observed for the CSP; its major decomposition occurred in three steps starting at 196.00, 305.10, and 428.24 °C, respectively. Specifically, the stage centered at 232.39 °C was caused presumably by the decomposition of the functionalized alpha-linolenic fatty acid chains. Furthermore, the main and biggest oil decomposition occurred between 305.10 and 428.24 °C, which was attributed to monounsaturated and saturated fatty acid decomposition, respectively. Finally, the last one had a weight loss centered at 435.68 °C, corresponding to the secondary thermal decomposition superimposed on the main reaction.

##### Physicochemical Characteristics of Vegetable Polyols

Moreover, we determined the acid value (AV), iodine vale (IV), saponification number (SN), kinematic viscosity, density, and OH numbers of the polyols, and the results are also included in Table 2.

The AV and SN for the polyol samples were higher when compared to the starting oils. It is important to mention that the CIP and CSP samples did not contain free acids, other than fatty acids; then, the acid value may be directly converted to percent free fatty acids. Hence, the slight increase in the AV and SN could be associated with the possible deterioration of the studied oils at the moment of producing polyols, such as rancidity. Dileesh et al. reported that the cause for rancidity is the hydrolytic or oxidative cleavage of triglycerides, causing the formation of free fatty acids in oils or fats [43]. The same behavior occurred with the values of kinematic viscosity and density, and both values are major, as expected. In the case of the viscosity, the values for CIP and CSP were 3637 and 5746 mm^2^/s, respectively. The big difference in these values were due to the hydrogen bonding, which was directly proportional to the major amount of OH groups present in the CSP (see OH functionality in Table 2). The obtained results showed a similarity tendency with the results reported by Knothe and Steidly [44]. In this study, the authors mentioned that free fatty acids or compounds with hydroxy groups possessed significantly higher viscosity than the starting material without hydroxy groups. Additional proof that the hydrolysis reaction of CIO and CSO had occurred was reflected in the iodine values of their respective polyols. While examining the iodine values for the CIP and CSP and their respective oils, the values decreased dramatically, which was evident since the unsaturation (C=C bonds) in the CIO and CSO were replaced by hydroxyl groups.

Furthermore, we determined the OH functionality, which is an important indicator of the crosslinking that can be obtained in future polyurethane foams. The OH numbers of the CIP and CSP were 225.0 and 240.8 mg KOH/g, respectively. The theoretical molecular weight (*M_w_*) shown in Table 2 for CIP and CSP (1122.4 and 1166.1 g/mol, respectively) was calculated from the composition of polyols, based on assumption that no oligomers were formed, and considering that one OH group was formed per double bond. The OH functionality results showed values of 4.50 and 5.00 for CIP and CSP, respectively. It is important to note that these differences between the OH values were due to the fatty composition of the starting oils. According to these values, we can expect the preparation of rigid foams [45]. Rigid polyurethane foams are widely used in engineering applications, such as thermal insulation, building materials, chemical pipelines, and space filling, among others [46].

Finally, we compared the results of polyols with other commercial polyols using the parameters described before, specifically OH functionality, such as epoxidized soybean (soy-H_2_) [47], organosolv lignin, and soda lignin-based polyols with OH functionality values of 3.5, 6.0, and 9.0, respectively [48,49,50]. These results show that our product could be used as a potential economical and environmentally friendly material to obtain rigid foams.

## 4. Conclusions

Two novel polyols were successfully synthesized from two non-edible vegetable oils, *C. integerrima* and *C. salicifolia*, by in situ epoxidation and hydroxylation of oils in a one single step. The physicochemical and spectroscopic analysis, presented above, successfully characterized the reactions of polyol formation. Specifically, the FTIR, TGA, and NMR spectroscopic results confirmed complete disappearance of the signature of the double bonds present in the vegetable oils, and the incorporation of hydroxyl groups to form their respective polyols. In addition, the iodine value, acid value, hydroxyl number, hydroxyl functionality, density, and viscosity of the synthesized polyols were also successfully determined. All these values were higher for CIP and CSP when compared to starting oils, with the exception of the iodine value, which decreased to near 0. Finally, the data collected in this study have established an essential starting point for the obtention of new materials. Specifically, we plan for a future study to examine the obtention of a new class of rigid polyurethane foams that could be used in engineering applications.

## Figures and Tables

**Table 1:** Chemical composition and characteristics of *C. integerrima* oil (CIO) and *C. salicifolia* oil (CSO).

Parameters	Oils	
	CIO	CSO
Fatty acid (%)		
Myristic, C_14:0_	0.06	0.04
Palmitic, C_16:0_	10.54	8.46
Palmitoleic, C_16:1_	0.05	0.08
Margaric, C_17:0_	0.00	0.00
Stearic, C_18:0_	2.03	2.06
Oleic, C_18:1_	23.50	15.08
Linoleic, C_18:2_	31.11	20.40
Gamma- linolenic, C_18:3_	0.48	0.00
Alpha-linolenic, C_18:3_	26.39	46.60
Gondoic, C_20:1_	5.35	6.34
Eicosadienoic C_20:2_	0.48	0.73
Cox value	9.35	12.24
AV (mg KOH/g oil)	0.17	0.25
IV (g I_2_/100 g oil)	143.8	179.3
PV (meq O_2_/kg oil)	19	18
SN (mg KOH/g oil)	196	194
Kinematic Viscosity (mm^2^/s)	58.6	48.8
Density (g/cm^3^)	0.884	0.896

**Table 2:** Chemical composition and characteristics of *C. integerrima* polyol(CIP) and *C. salicifolia* polyol (CSP).

Parameters	Polyols	
	CIP	CSP
AV (mg KOH/g)	4.50	12.95
IV (g I_2_/100 g)	0.2	0.5
SN (mg KOH/g)	227	235
Kinematic Viscosity (mm^2^/s)	3637	5746
Density (g/cm^3^)	1.012	0.921
Molar Mass (g/mol)	1122.4	1166.1
OH numbers (mg KOH/g)	225.0	240.8
OH functionality	4.50	5.00
